# Plant-Produced N-glycosylated Ag85A Exhibits Enhanced Vaccine Efficacy Against *Mycobacterium tuberculosis* HN878 Through Balanced Multifunctional Th1 T Cell Immunity

**DOI:** 10.3390/vaccines8020189

**Published:** 2020-04-18

**Authors:** Hongmin Kim, Kee Woong Kwon, Jaehun Park, Hyangju Kang, Yongjik Lee, Eun-Ju Sohn, Inhwan Hwang, Seok-Yong Eum, Sung Jae Shin

**Affiliations:** 1 Department of Microbiology, Institute for Immunology and Immunological Diseases, Brain Korea 21 PLUS Project for Medical Science, Yonsei University College of Medicine, Seoul 03722, Korea; goldhm@yuhs.ac (H.K.); KKEEWEE@yuhs.ac (K.W.K.); jhpark8908@yuhs.ac (J.P.); 2 BioApplications Inc., Pohang 37668, Korea; skyline2@postech.ac.kr (H.K.); yjlee@postech.ac.kr (Y.L.); ejsohn@postech.ac.kr (E.-J.S.); 3 School of Interdisciplinary Bioscience and Biotechnology, Pohang University of Science and Technology, Pohang 37673, Korea; 4 Division of Integrative Biosciences and Biotechnology, Pohang University of Science and Technology, Pohang 37673, Korea; ihhwang@postech.ac.kr; 5 Division of Immunopathology and Cellular Immunology, International Tuberculosis Research Center, Changwon 51755, Korea; syeumkr@gmail.com

**Keywords:** *Mycobacterium tuberculosis*, *Nicotiana benthamiana*, glycosylation, Ag85A, Th1 response, subunit vaccine, vaccine antigen

## Abstract

Tuberculosis (TB) is one of the deadliest infectious diseases worldwide and is caused by *Mycobacterium tuberculosis* (Mtb). An effective vaccine to prevent TB is considered the most cost-effective measure for controlling this disease. Many different vaccine antigen (Ag) candidates, including well-known and newly identified Ags, have been evaluated in clinical and preclinical studies. In this study, we took advantage of a plant system of protein expression using *Nicotiana benthamiana* to produce N-glycosylated antigen 85A (G-Ag85A), which is one of the most well-characterized vaccine Ag candidates in the field of TB vaccines, and compared its immunogenicity and vaccine efficacy with those of nonglycosylated Ag85A (NG-Ag85A) produced with an *Escherichia coli* system. Notably, G-Ag85A induced a more robust IFN-γ response than NG-Ag85A, which indicated that G-Ag85A is well recognized by the host immune system during Mtb infection. We subsequently compared the vaccine potential of G-Ag85A and NG-Ag85A by evaluating their immunological features and substantial protection efficacies. Interestingly, G-Ag85A yielded moderately enhanced long-term protective efficacy, as measured in terms of bacterial burden and lung inflammation. Strikingly, G-Ag85A-immunized mice showed a more balanced proportion of multifunctional Th1-biased immune responses with sustained IFN-γ response than did NG-Ag85A-immunized mice. Collectively, plant-derived G-Ag85A could induce protective and balanced Th1 responses and confer long-term protection against a hypervirulent Mtb Beijing strain infection, which indicated that plant-produced G-Ag85A might provide an excellent example for the production of an Mtb subunit vaccine Ag and could be an effective platform for the development of anti-TB vaccines.

## 1. Introduction

Tuberculosis (TB), which is caused by *Mycobacterium tuberculosis* (Mtb), remains a major infectious threat with high morbidity and mortality worldwide [1], and as a result, researchers continually aim to develop effective vaccines against TB. At present, the *Mycobacterium bovis* Bacillus Calmette-Guérin (BCG) vaccine is the only prophylactic vaccine used, but the insufficient pulmonary protection that BCG provides against TB means that the development of effective novel vaccines is urgently needed [2]. Various types of adjuvants, antigen (Ag) targets and vaccine platforms have been developed to improve the Mtb vaccine [3,4]. These efforts have yielded many results, some of which include optimistic outcomes in the clinical phase, but more diverse and dynamic pipelines are needed [5].

In 2018, two multi-Ag subunit vaccines against TB that induce Ag-specific multifunctional CD4^+^ T cell responses demonstrated promising results in clinical efficacy trials [6,7]. These vaccine candidates contain highly immunogenic Ags, such as PPE18 and Ag85B [6,7]. Therefore, the identification and production of promising vaccine Ags are the first and most crucial steps in the development of TB vaccines. Ags could be produced for vaccines using several approaches that have specific characteristics based on the system (bacteria, yeast, insect cells, and plants). The bacterial expression system produces recombinant Ags with a high yield and low cost, but the quality of Ags in terms of modification and solubility might not be appropriate [8]. The yeast expression system is safe but produces a low yield [9]. Insect cells can express proteins at high levels and with proper modification, but continuous expression is limited [10]. Meanwhile, plants have become a promising platform for the production of protein pharmaceuticals due to their safety and cost effectiveness and the easy scalability of the products. First, plants are a safer production system than animal cells because plants cannot be contaminated by animal pathogens such as viruses and bacteria or prions [11]. Second, plant systems are highly scalable, and their infrastructure requires a low capital investment [12,13]. Therefore, plant systems are potential appropriate platforms for vaccine development. Indeed, many previous studies on the production of antibodies (Abs), vaccines, and protein therapeutics in plants have introduced and advanced this field. Plant-derived Abs were produced for therapy and passive immunization targeted to human immunodeficiency virus [14], B-cell lymphoma [15], rabies virus [16], and anthrax toxin [17]. For vaccine development, virus-like particles that display Zika virus envelope protein domain III were produced quickly from *Nicotiana benthamiana* and easily purified in large quantities [18]. In the field of TB, various plant systems have been utilized to express the Ags of Mtb [19,20], and these studies have resulted in BCG booster vaccines and protein therapeutics with a high immunogenicity that promote increased cellular and humoral immune responses as well as reduced bacterial burden [21,22].

Beyond the selection of Ags, the posttranslational modifications of Ags, which mimic the authentic nature of Ags, have been investigated to develop more effective vaccines against many infectious diseases. One such approach involves investigating the relationship between Ag glycosylation and vaccine effectiveness [23,24]. Polysaccharide conjugation to carrier proteins promotes the production of specific Abs in the immune system [25]. Moreover, CD4^+^ and CD8^+^ T cell responses are significantly increased by increasing Ag uptake by dendritic cells in a manner dependent on the carbohydrate modifications of the ovalbumin (OVA) protein [26]. These studies suggest the possibility that the efficacy of a vaccine can be increased by the glycosylation of Ags, which indicates that a plant expression system in which glycosylation occurs can potentially be used for the development of vaccines instead of the *Escherichia coli* expression system. In this regard, plants have the advantage of being able to produce proteins with posttranslational modifications, such as N- or O-glycosylation.

The Ag85 complex is a 30–32 kDa family of three closely related proteins (Ag85A, Ag85B, and Ag85C) with enzymatic mycolyl-transferase activity that are involved in the biogenesis of cord factor and in the coupling of mycolic acids to arabinogalactan in cell walls [27]. Members of the Ag85 family exhibit strong potential to induce a Th1-type immune response, which is important for the regulation of intracellular infection and are thus one of the most promising candidates for TB vaccine Ags. In particular, Ag85A and Ag85B, which were initially purified from BCG and Mtb culture filtrate, respectively, induce strong T cell proliferation and IFN-γ production in most healthy individuals latently infected with Mtb and in BCG-vaccinated mice and humans but not in TB patients [28,29]. These results are sufficient for the selection of Ag85A as a prominent candidate of the Mtb vaccine; therefore, Ag85A has been studied for various approaches toward an Mtb vaccine, such as a modified vaccinia Ankara virus expressing Ag85A (MVA85A), Ag85A-overexpressing BCG or DNA vaccines [30,31,32]. These studies demonstrate the proven efficacy of Ag85A as a TB vaccine Ag, suggesting that it can be an appropriate candidate for comparing the efficacy of vaccines produced by different expression systems. 

Despite the many advantages and possibilities of plant-based expression systems, there are not many substantial studies of the efficacy of TB vaccines produced with plant systems. Therefore, we focused on the benefits of the plant expression system for the development of new effective TB vaccines and used Ag85A as a prominent target of the Mtb vaccine. In the present study, we produced N-glycosylated Ag85A (G-Ag85A) in *N. benthamiana* and compared the protective vaccine efficacy and immunogenicity of G-Ag85A with those of nonglycosylated recombinant Ag85A (NG-Ag85A). The immunogenicity and the potential protective efficacy of G-Ag85A and NG-Ag85A were assessed by directly comparing the responses to Mtb HN878 challenge in a mouse model following the delivery of these Ags as subunit vaccines.

## 2. Materials and Methods

### 2.1. Experimental Animals and Ethics Statement

Specific-pathogen-free 6- to 8-week-old female wild-type C57BL/6 mice were purchased from SLC Inc. (Shizuoka, Japan) and strictly maintained under barrier conditions in a BSL-3 facility at the Avison Biomedical Research Center at Yonsei College of Medicine. The experimental protocols used in this study were reviewed and approved by the Ethics Committee and Institutional Animal Care and Use Committee (Permit Number: 2017-0264) of the Laboratory Animal Research Center at Yonsei University College of Medicine (Seoul, Korea). All animal experiments were performed according to the Korean Food and Drug Administration (KFDA) guidelines and regulations.

### 2.2. Abs and Reagents

A LIVE/DEAD™ Fixable Near-IR Dead Cell Stain Kit was purchased from Molecular Probes (Carlsbad, CA, USA). The following Abs were used for flow cytometry analyses: phycoerythrin (PE)-conjugated monoclonal antibody (mAb) against IFN-γ, violet 450-conjugated mAb against CD44, allophycocyanin (APC)-conjugated mAb against TNF-α, brilliant violet (BV) 605-conjugated mAb against Thy1.2, BV711-conjugated mAb against CD8, and PerCP-Cy5.5-conjugated mAb against CD4; these were purchased from BD Bioscience (San Jose, CA, USA), and Alexa Fluor 700-conjugated mAb against CD62L and PE-Cy7-conjugated mAb against IL-2 were purchased from eBioscience (San Diego, CA, USA). The CAF01 liposome adjuvant was kindly provided by the Statens Serum Institut (SSI, Demark).

### 2.3. Mtb Strain and Culture Conditions

The Mtb HN878 strain was collected from the Korean Tuberculosis Research Institute (KIT, Osong, Chungcheongbuk-do, Korea), and BCG (Pasteur 1173P2) was obtained from the Pasteur Institute (Paris, France). The mycobacterial strains used in this study were cultured as previously described [33]. The seed lots of each strain were maintained in small aliquots at −80 °C until use. After confirmation of the predominant presence of single cells in the final preparation based on acid-fast staining, the number of colony-forming units (CFUs) per 1 mL of each seed lot on a 7H10 agar plate was measured using a viable cell counting assay, and the cells were then used for subsequent experiments.

### 2.4. Expression and Purification of Ag85A

For the expression of NG-Ag85A, we expressed the protein using the *E. coli* expression system as previously reported [34]. To produce the glycosylated Ag85A protein, *N. benthamiana* was used for Ag85A expression as described below.

### 2.5. Plasmid Construction and Transient Expression

For the expression of Ag85A in *N. benthamiana*, a recombinant construct, *1300-HCH:Ag85A*, was generated as described below. The first Ag85A coding sequence was codon-optimized for efficient expression in *N. benthamiana* and was chemically synthesized. A DNA fragment containing Ag85A together with an enterokinase cleavage site was amplified by polymerase chain reaction (PCR) using the primers Ag85A-F and Ag85A-R (Table S1). The forward primer also contained the enterokinase cleavage site and *Xma*I restriction site, and the reverse primer included an ER retention signal, HDEL. A DNA fragment encoding CBM3, which was used as an affinity purification tag, was prepared from p1300-C3bdSU-hIL6 by PCR using the gene-specific primers CBM3-F and CBM3-R [35]. The forward primer included a hemagglutinin (HA) epitope and the *Bam*HI restriction site, and the reverse primer included an HL (helical linker) and the *Xma*I restriction site. PCR amplifications were conducted using Ex Taq polymerase (Takara Bio, Kusatsu, Japan) in 25 cycles of denaturation at 94 °C (30 s), annealing at 53 °C (30 s), and extension at 72 °C (90 s for Ag85A, 40 s for CBM3). To generate the recombinant construct *1300-HCH:Ag85A*, the two PCR fragments were then inserted in the order of *CBM3* and *Ag85A* into the sequence downstream of BiP in the pCAMBIA 1300 binary vector containing the double enhancer-containing CaMV 35S promoter (d35S), BiP leader sequence, and HSP terminator using the restriction endonucleases *BamH*I with *Xma*I and *Xma*I with *Sac*I, respectively.

For the expression of recombinant Ag85A, the expression vector *1300-HCH:Ag85A* was introduced into *Agrobacterium tumefaciens* strain LBA4404 by electroporation using MicroPulser (Bio-Rad Laboratories, Hercules, CA) according to the manufacturer’s protocol. The gene silencing suppressor p38 construct was cotransformed to enhance the expression of fusion protein by suppressing gene silencing [35]. For the cotransformation of *1300-HCH:Ag85A* and p38, two *Agrobacterium* each harboring *1300-HCH:Ag85A* and p38 were collected by centrifugation at 3500 g for 15 min and resuspended in infiltration buffer (10 mM MES, 10 mM MgSO4, and 100 μM acetosyringone, pH 5.6). Two *Agrobacterium* suspension were mixed at a 1:1 (v/v) ratio, and the mixture was adjusted to obtain an OD_600_ of 1.0. The 5- to 6-week-old *N*. *benthamiana* plants were subsequently transformed with *Agrobacterium* mixture by vacuum infiltration [36]. The infected leaves were harvested four days after infiltration.

### 2.6. Purification of Recombinant G-Ag85A from the Leaf Extracts of N. Benthamiana

The harvested leaves (20 g) were frozen in liquid nitrogen and ground into powder using a mortar and pestle. Total protein extracts were prepared using five volumes (w/v) of extraction buffer (50 mM Tris-Cl, 150 mM NaCl, 0.2% Triton X-100, and protease inhibitor, pH 7.2). The mixture was centrifuged at 20,000× *g* for 30 min at 4 °C, and the supernatant was loaded into a column filled with microcrystalline cellulose (MCC; 3 g) at a flow rate of 100 μL/min. The column was washed with three column volumes of washing buffer (50 mM Tris-Cl and 150 mM NaCl, pH 7.2) at a flow rate of 1 mL/min. The recombinant Ag85A fusion protein-bound cellulose beads were released from the column, precipitated by centrifugation at 2000× *g* and 4 °C for 10 min and resuspended in 10 mL of enterokinase reaction buffer (50 mM Tris-Cl, 150 mM NaCl, and 1 mM CaCl_2_, pH 7.2). Enterokinase (5 unit/μL; NBM Inc., Iksan, Korea) was added to the recombinant Ag85A fusion protein-bound cellulose beads and incubated at 28 °C for 4 h. The soluble fraction containing G-Ag85A was collected after centrifugation at 2000× *g* and 4 °C for 10 min, and enterokinase was removed by affinity chromatography using STI-Sepharose. Finally, the flow-through fraction from the affinity chromatography step that contained G-Ag85A was separately collected. Purified plant-produced Ag85A was analyzed by 10% SDS-PAGE. In addition, protein samples were saved from each fraction, including the total fraction, and subjected to western blot analysis using anti-Ag85A Ab (Abcam, Cambridge, MA, USA) to check the expression of fusion protein and efficiencies of the MCC bead binding and enterokinase-mediated cleavage reactions.

### 2.7. Deglycosylation of Plant-Produced Ag85A

Purified plant-produced Ag85A was treated with Endo-H (New England Biolabs, Beverly, MA, USA) or PNGase F (New England Biolabs, Beverly, MA, USA) according to the manufacturer’s recommended protocols. Ag85A (1 μg) was added to 1 μL of 10X glycoprotein denaturing buffer (5% SDS, 400 mM DTT), and distilled water was added to the mixture to obtain a reaction volume of 10 μL. The mixture was then incubated in boiling water for 10 min for protein denaturation and briefly cooled on ice. Subsequently, 2 μL of 10X glycobuffer 3 (500 mM sodium acetate, pH 6), 1 μL of Endo-H, and 7 μL of distilled water were added, and the reaction mixture was incubated at 37 °C for 1 h. For PNGase F treatment, Ag85A (1 μg) was added to 1 μL of 10X glycoprotein denaturing buffer, and distilled water was added to the mixture to obtain a reaction volume of 10 μL. The Ag85A protein was denatured by incubation in boiling water for 10 min, and the sample was then chilled on ice. The reaction volume was increased to 20 μL through the addition of 2 μL of 10X glycobuffer 2 (500 mM sodium phosphate, pH 7.5), 2 μL of 10% NP-40, 5 μL of distilled water, and 1 μL of PNGase F, and the resulting mixture was incubated at 37 °C for 1 h. Both samples were analyzed by western blotting using anti-Ag85A polyclonal Ab (Abcam, Cambridge, MA, USA).

### 2.8. Immunization of Mice with Individual Ag85A and Challenge with Mtb Beijing Strain HN878

C57BL/6J female mice were subcutaneously immunized once with 2 × 10^5^ CFUs of BCG Pasteur 1173P2. For the subunit vaccine immunization, mice were immunized three times at 2-week intervals via subcutaneous injection. Each immunization contained 2 μg of NG-Ag85A or G-Ag85A protein adjuvanted with CAF01 liposomes. The mice in the control group were immunized with only CAF01. Four weeks after the last immunization, spleen and lung cells were prepared and analyzed in terms of their immunogenicity (*n =* 4–6 mice/group). Six weeks after the final immunization, the adjuvant control group and the vaccinated groups (BCG, NG-Ag85A, and G-Ag85A) were infected with the Mtb Beijing strain HN878 at approximately 70 CFUs per mouse via aerosol (*n =* 10–12 mice/group; 5–6 mice/group for each sacrifice time point). Specifically, the mice were exposed to the HN878 strain for 60 min in the inhalation chamber, which had been previously calibrated to deliver a predetermined dose (Glas-Col, Terre Haute, IN, USA). One day after infection, the infected mice were sacrificed to confirm the initial bacterial burden (*n =* 3). Four weeks and twelve weeks after challenge, mice were sacrificed to measure the efficacy of vaccination.

### 2.9. Splenocyte and Lung Cell Preparation

Single-cell suspensions from the lungs and spleen were prepared as follows. The spleens and lungs from mice in each group were minced into 2–4 mm pieces using scissors. The lung tissue was incubated in 3 mL of cellular dissociation buffer (RPMI medium (Biowest, Nuaillé, France) containing 0.1% collagenase type IV (Worthington Biochemical Corporation, NJ, USA) and 1 mM CaCl_2_ and 1 mM MgCl_2_) for 30 min at 37 °C. Splenocytes and lung cells were filtered via a 40 μm cell strainer (BD Bioscience, San Diego, CA, USA) in RPMI medium supplemented with 2% fetal bovine serum (FBS, Biowest) using a sterile 10-mL syringe. The erythrocytes were lysed using red blood cell lysis buffer (Sigma–Aldrich, St. Louis, MI, USA) for 3 min at room temperature, and then single cells were washed twice with RPMI medium supplemented with 2% FBS.

### 2.10. Cytokine Measurement

Single cells from the lungs and spleens of Mtb-infected or immunized mice were stimulated with Ag proteins (NG-Ag85A or G-Ag85A) for 12 h at 37 °C. The levels of secreted IFN-γ in the culture supernatant were measured using a commercial ELISA kit according to the manufacturer’s instructions (BD Bioscience, San Jose, CA, USA).

### 2.11. Ab Titer Measurement in Serum

The levels of NG-Ag85A- or G-Ag85A-specific total immunoglobulin G (IgG), IgG1, and IgG2c in serum were evaluated as Ag-specific type 1 or type 2 immune responses. Briefly, 96-well plates were coated with 1 μg/mL NG- or G-Ag85A. ESAT-6 was used for negative control. After incubation of the diluted serum, horseradish peroxidase (HRP)-conjugated Ab against total IgG, IgG1 (BD Bioscience, San Diego, CA, USA) or IgG2c (Southern Biotech, Birmingham, AL, USA) was used as a secondary Ab. The optical densities (ODs) were determined at 450 nm.

### 2.12. Intracellular Cytokine Staining

Individual lung and spleen cells were prepared from immunized and Mtb-infected mice and stimulated with 2.5 µg/mL NG- or G-Ag85A at 37 °C for 12 h in the presence of GolgiPlug and GolgiStop (BD Bioscience, San Jose, CA, USA). First, the cells were washed with 1X PBS (pH 7.4), and the Fc receptor was blocked with an anti-CD16/32-blocking Ab at 4 °C for 15 min. Surface molecules were stained with fluorochrome-conjugated Abs against Thy1.2, CD4, CD8, CD44, and CD62L, and the LIVE/DEAD^TM^ Fixable Dead Cell Kit was used for 30 min at 4 °C. The cells were then washed with PBS, fixed, and permeabilized with Cytofix/Cytoperm (BD Biosciences) for 30 min at 4 °C. The permeabilized cells were washed twice with Perm/Wash (BD Biosciences) and stained with PE-conjugated anti-IFN-γ, APC-conjugated anti-TNF-α, and PE-Cy7-conjugated anti-IL-2 Abs for 30 min at 4 °C. The cells were washed twice with Perm/Wash and fixed with IC fixation buffer (eBioscience, San Diego, CA, USA) for flow cytometry analysis.

### 2.13. Analysis of Histopathology and Mtb Burden

The protective efficacy of the various vaccination strategies was determined through analysis of the histopathology and bacterial growth in the lung and spleen. The mice from every vaccinated group were sacrificed at 4 and 12 weeks after Mtb infection to evaluate the protective efficacies of each vaccination. For the lung histopathology analysis, the right superior lobes were preserved overnight in 10% formalin and embedded in paraffin. The lung was sectioned at 4–5 μm, stained with H&E, and evaluated by light microscopy. For the bacterial growth analysis, the lung and spleen were homogenized, and serially diluted samples were plated onto Middlebrook 7H11 agar plates (Becton Dickinson, Franklin Lakes, NJ, USA) supplemented with 10% OADC (Difco Laboratories, Detroit, Mich.), 2 μg/mL 2-thiophenecarboxylic acid hydrazide (Sigma–Aldrich, St. Louis, MO, USA), and amphotericin B (Sigma–Aldrich). After incubation at 37 °C for 3–4 weeks, the bacterial colonies were counted.

### 2.14. Statistical Analyses

The statistical analyses were conducted using GraphPad Prism V5.0 (GraphPad Software, San Diego, CA, USA). The differences between two groups were analyzed using an unpaired Student’s *t*-test. One-way ANOVA followed by Tukey’s multiple comparison tests was used to analyze data from more than two groups. All the values are expressed as the means (± standard deviations, SDs). Statistical significance was determined at * *p* < 0.05, ** *p* < 0.01 or *** *p* < 0.001.

## 3. Results

### 3.1. Expression and Purification of Glycosylated Ag85A in N. Benthamiana

**Figure 1:**
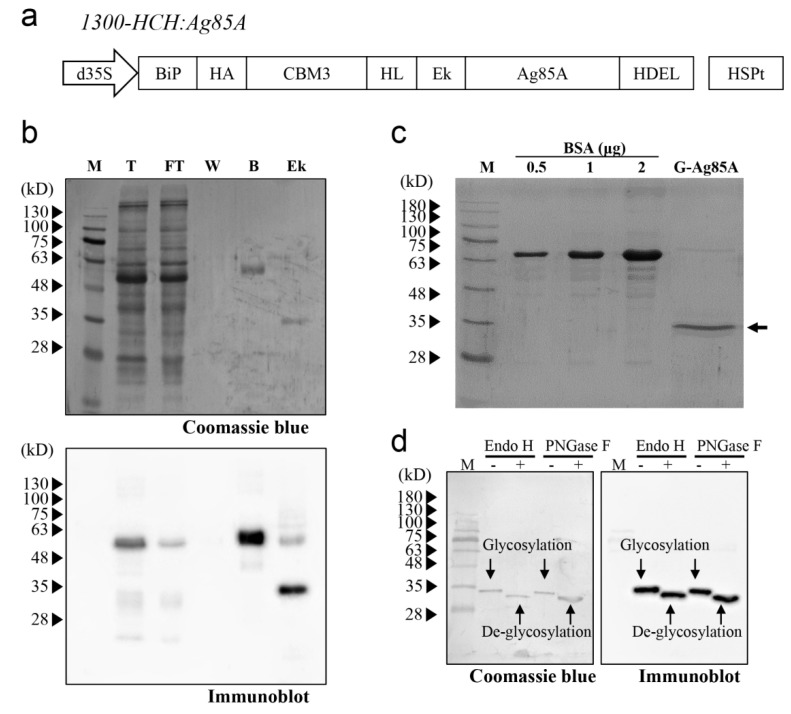
Expression and purification of Ag85A in *N. benthamiana*. (**a**) Schematic representation of the recombinant Ag85A fusion construct. The fusion construct contains a BiP signal peptide, HA tag, CBM3, helical linker (HL), enterokinase cleavage site (Ek), Ag85A, and ER retention signal HDEL. This fusion construct was under the control of the cauliflower mosaic virus containing two enhancers, a 35S promoter and a heat shock protein (HSP) terminator. (**b**) Binding of the recombinant Ag85A fusion protein to MCC beads and enterokinase-mediated cleavage. The protein samples for each fraction were analyzed by western blotting using an anti-Ag85A antibody. M, protein standard marker; T, total; FT, flow-through; W, washing; B, MCC beads; Ek, enterokinase-treated supernatant and beads. (**c**) Western blot analysis of purified G-Ag85A. The purified G-Ag85A protein was analyzed by SDS-PAGE. BSA was loaded together to check the amount of G-Ag85A protein. (**d**) Deglycosylation of G-Ag85A. Purified G-Ag85A was treated with Endo-H or PNGase F and subjected to SDS-PAGE together with untreated G-Ag85A and then to western blot analysis using an anti-Ag85A antibody.

We decided to use *N*. *benthamiana* as the host for the production of Ag85A in plants, and we compared the Ag85A produced in *N*. *benthamiana* with that expressed in *E. coli*. Accordingly, Ag85A was codon-optimized to *N*. *benthamiana*. Specifically, for the production of recombinant Ag85A in plants, we designed a recombinant Ag85A fusion construct, *1300-HCH:Ag85A* (Figure 1a). The construct contained an ER leader sequence that targeted the ER, an HA-tagged CBM3 domain for cellulose-based affinity purification, an HL followed by a enterokinase site to remove the N-terminal region of Ag85A, an Ag85A-coding region, and an ER retention signal to induce accumulation of the recombinant protein in the ER [35,37,38,39]. An HL was introduced between CBM3 and the enterokinase cleavage site to facilitate the access of enterokinase to the target site. HA was added at the N terminus of CBM3 to detect the recombinant Ag85A fusion protein by western blotting using an anti-HA Ab (Figure 1a). Based on the abovementioned design for the Ag85A expression cassette, in vitro enterokinase treatment would produce Ag85A with no additional amino acid residues except the C-terminal HDEL, an ER retention signal [40].

To express the recombinant Ag85A fusion construct in *N. benthamiana*, the fusion construct *1300-HCH:Ag85A* was introduced into *Agrobacterium* strain LBA4404. Subsequently, the fusion construct was introduced into *N. benthamiana* leaves via *Agrobacterium*-mediated transformation [35,36]. Ag85A protein was purified using protein extracts at four days after infiltration, and protein samples from each fraction during purification were analyzed by western blotting. First, the full-length fusion protein was detected at the expected size, indicating that the fusion construct was expressed properly in *N. benthamiana* (Figure 1b). The majority of the full-length recombinant Ag85A fusion proteins were bound to the MCC beads, and only a minor portion was detected in the flow-through fraction. After treatment with enterokinase, the molecular mass of the Ag85A-specific protein band changed from 60 kDa to 35 kDa (Figure 1b), which indicated that enterokinase cleavage removed the N-terminal domain of the plant-produced recombinant Ag85A fusion protein. After removing enterokinase by using STI-Sepharose, the majority of the proteins in the flow-through fraction were approximately 35 kDa, although a small portion of the proteins was 70 kDa (Figure 1c). Both the 35-kDa and 70-kDa proteins were detected in the western blot analysis with anti-Ag85A Ab, which indicated that these proteins were indeed Ag85A and that the upper band was potentially a dimer of Ag85A (Figure S1). The yield of the 35-kDa protein was 22.0 μg/g fresh weight of infiltrated leaves.

The size of the plant-produced Ag85A with a molecular mass of 35 kDa was slightly larger than the expected size, which indicated the possibility of posttranslational modification. In this study, the recombinant Ag85A fusion protein was targeted to the ER. In addition, this protein contained a potential N-glycosylation site (Asn-Asn-Thr) at position 203 of mature Ag85A and has been reported to be N-glycosylated when expressed in the ER of mammalian cells [41]. To investigate whether plant-produced Ag85A is N-glycosylated in plants, we added two enzymes that can remove N-glycans from proteins, endoglycosidase H (Endo-H) and PNGase F [42]. Both enzymes increased the migration of Ag85A proteins in the SDS/PAGE gel, which indicated that plant-produced Ag85A was N-glycosylated in plants (Figure 1d). In addition, the N-glycan of plant-produced Ag85A was expected to be a high-mannose type because it was targeted to the ER and cleaved by Endo-H. In contrast, it was confirmed that *E. coli*-produced Ag85A was not sensitive to Endo-H (Figure S2). Thus, we named the plant-produced Ag85A as G-Ag85A, to highlight the N-glycosylated characteristic of this protein while *E. coli*-produced Ag85A is named as NG-Ag85A.

### 3.2. Glycosylation of Ag85A Induced an Augmented Ag-specific IFN-γ response during Mtb Infection and Increased T Cell Proliferation in Coculture with Bone Marrow-Derived Dendritic Cells

**Figure 2:**
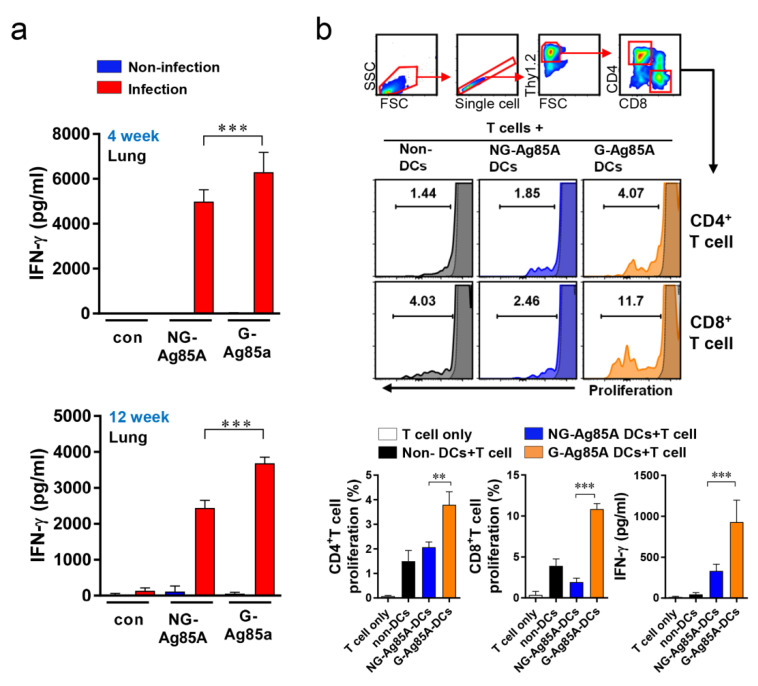
Comparison of IFN-γ production induced by plant-produced G-Ag85A and bacteria-produced NG-Ag85A stimulation and proliferation of T cells cocultured with BMDCs stimulated with G- and NG-Ag85A. (**a**) Mice were infected with approximately 70 CFUs of the Mtb Beijing strain HN878 per mouse via the aerosol route. Four and 12 weeks after the Mtb challenge, the infected or uninfected mice were sacrificed, and their lung cells (2 × 10^6^ cells) were stimulated with NG- or G-Ag85A (2.5 μg/mL) for 12 h at 37 °C. The concentrations of IFN-γ in the supernatant were measured using commercial ELISA kits. The data are presented as the mean ± SDs from 5–6 mice in each group. One-way ANOVA was used to determine the significance of the differences. *** *p <* 0.001. (**b**) BMDCs were treated with 5 μg/mL NG- or G-Ag85A for 24 h, and T cells were isolated from the spleens of Mtb-infected mice using a magnetic cell sorting system and labeled with violet proliferation dye 450. Isolated T cells were cocultured with the harvested BMDCs, and the proliferation of CD4^+^ T cells and CD8^+^ T cells was analyzed by flow cytometry. The concentrations of IFN-γ in the supernatant were measured using commercial ELISA kits. The displayed means ± SDs represent the data from two independent experiments. One-way ANOVA was used to determine the significance of the differences. ** *p <* 0.01, *** *p <* 0.001.

To determine the potential of an Ag as a vaccine target, the IFN-γ response could be used as a marker for Ag recognition because IFN-γ is a key Th1 CD4^+^ T cell and is important for protecting the host against Mtb [43]. Thus, we measured the recognition of G-Ag85A and NG-Ag85A in Mtb-infected mice. First, Mtb-infected mice were sacrificed at 4 and 12 weeks postinfection, cells from their lungs were separately stimulated with both types of Ag85A, and the levels of secreted IFN-γ were measured by ELISA (Figure 2a). The results showed that both NG-Ag85A and G-Ag85A induced IFN-γ secretion in lung cells, but more IFN-γ secretion from lung cells was induced by G-Ag85A than by NG-Ag85A. Bone marrow-derived dendritic cells (BMDCs) were stimulated with 5 μg/mL G- or NG-Ag85A and cocultured for three days with T cells from the spleens of Mtb-infected mice (Figure 2b). Consequently, G-Ag85A-stimulated BMDCs promoted increased proliferation of CD4^+^ and CD8^+^ T cells compared with that induced by NG-Ag85A-stimulated BMDCs. These results indicated that Ag85A is well recognized by the host immune system at the early and late infection phases and that G-Ag85A induces different immunogenic properties compared with those induced by NG-Ag85A.

### 3.3. Vaccine Experimental Design and Immunogenic Response in the Lung and Spleen of G-Ag85A- and NG-Ag85A-Immunized Mice

**Figure 3:**
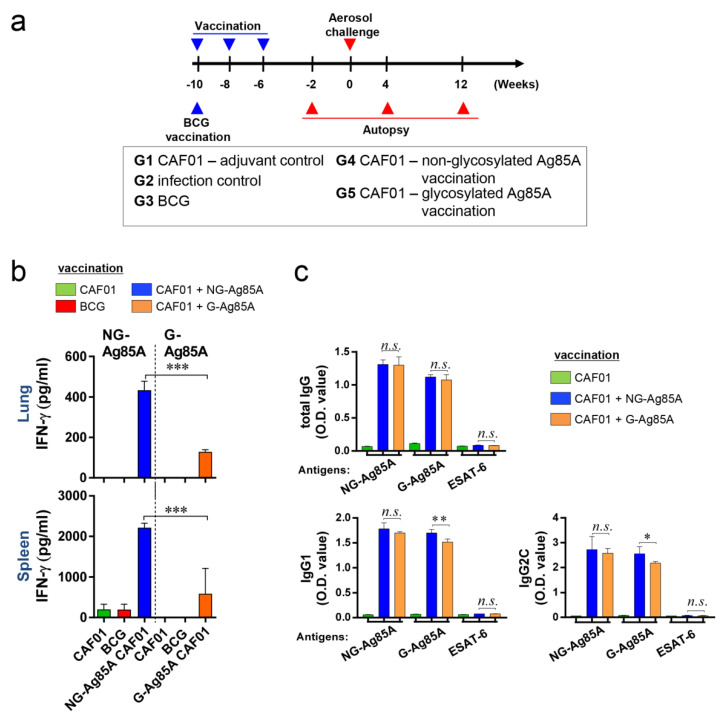
Comparison of the immunogenicity of plant-produced G-Ag85A and bacteria-produced NG-Ag85A in the lungs and spleens of immunized mice. (**a**) The immunizations were performed three times at 2-week intervals. Six weeks after the final immunization, the mice were challenged with Mtb via the aerosol route (*n* = 5–6/group). The mice were sacrificed 2 weeks before the challenge and 4 and 12 weeks after infection. (**b**) The lungs and spleens were removed, and 2 × 10^6^ spleen cells (splenocytes) or 1 × 10^6^ cells (lung cells) were cultured in microtiter plates and incubated with 2.5 μg/mL NG- or G-Ag85A for 12 h at 37 °C. The IFN-γ concentrations in the supernatant were detected by ELISA. (**c**) The NG-Ag85A- or G-Ag85A-specific IgG1, IgG2c, and total IgG levels in mouse serum were measured by ELISA. The data are presented as the mean ± SDs from 5–6 mice in each group. One-way ANOVA was used to determine the significance of the differences. * *p* < 0.05, ** *p <* 0.01, *** *p <* 0.001; *n.s.*, not significant.

To evaluate the efficacy of G-Ag85A and NG-Ag85A, we vaccinated mice with BCG, G- or NG-Ag85A. CAF01 was used as an adjuvant for G- or NG-Ag85A vaccination. Four weeks after the final immunization, the CAF01-, BCG-, CAF01/NG-Ag85A-, and CAF01/G-Ag85A-immunized groups were sacrificed for analysis (Figure 3a). The lungs and spleen were removed and used for the preparation of single-cell suspensions. These suspensions were then treated with 2.5 µg/mL G- or NG-Ag85A for 12 h, and the amount of IFN-γ secreted from the lung and spleen cells was then measured (Figure 3b). Unlike the postinfection results (4 and 12 weeks), the level of IFN-γ obtained with the NG-Ag85A treatment was higher than that obtained with the G-Ag85A treatment, and this finding was acquired with both the lung and spleen cells. The levels of total IgG, IgG1, and IgG2c in serum against NG-Ag85A, G-Ag85A or ESAT-6 were also detected by ELISA (Figure 3c). Abs generated by the immunization with G-Ag85A or NG-Ag85A showed specificity to both forms of Ag85A regardless of glycosylation. However, no specificity was shown against ESAT-6. Every type of IgG was well detected, and IgG1 and IgG2c from NG-Ag85A immunized mice tended to show higher responses against G-Ag85A than IgG1 and IgG2c from G-Ag85A-immunized mice. These results indicated that both G- and NG-Ag85A have immunogenic potential.

### 3.4. G-Ag85A Immunization Induced an Increased Number of CD4^+^ Multifunctional Th1 Cells Compared to NG-Ag85A Immunization Prior to Mtb Challenge

**Figure 4:**
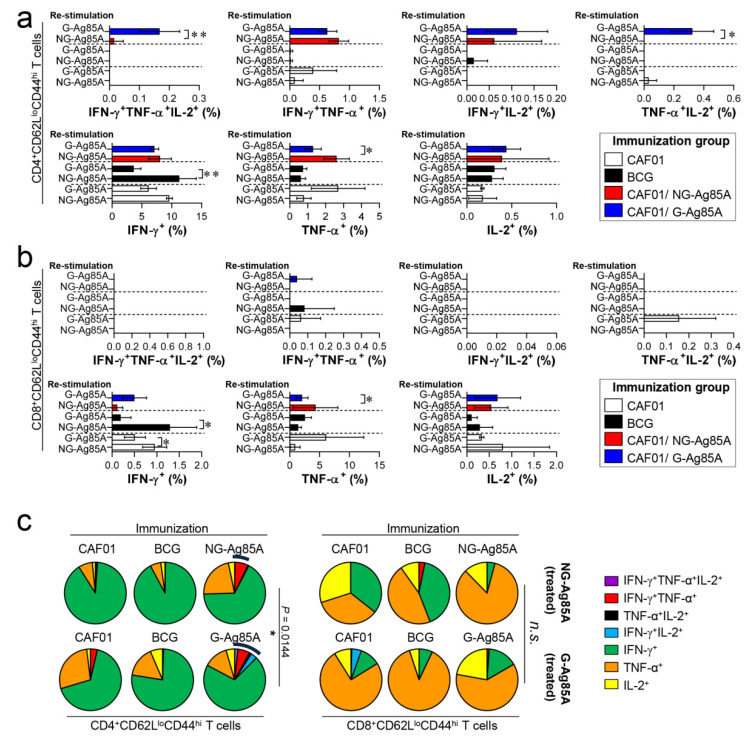
Comparison of the induction of Ag-specific multifunctional T cells in mice immunized with plant-produced G-Ag85A and bacteria-produced NG-Ag85A. Four weeks after the final immunization, the mice from each group were sacrificed, and their lung cells (1 × 10^6^ cells) were restimulated ex vivo with NG-Ag85A or G-Ag85A (2.5 μg/mL). The percentages of Ag-specific CD4^+^CD62L^lo^CD44^hi^ and CD8^+^CD62L^lo^CD44^hi^ T cells producing IFN-γ, TNF-α, and/or IL-2 among the cells isolated from the lungs of each group of mice were analyzed via flow cytometry by gating the cells into CD4^+^ (**a**) and CD8^+^ (**b**) T cells. The data are presented as the mean ± SDs from 5–6 mice in each group. One-way ANOVA was used to determine the significance of the differences. * *p <* 0.05, ** *p <* 0.01, and *** *p <* 0.001. (**c**) The mean frequencies of cells coexpressing IFN-γ, TNF-α, and/or IL-2 are shown in the pie charts. The arcs around the pie charts indicate the percentage of T cells that produced multiple cytokines. Unpaired *t*-tests were used to determine the significance of the differences in the percentages of polyfunctional T cells between the G-Ag85A- and NG-Ag85A-immunized groups.

The composition of functional CD4^+^ and CD8^+^ T cells is associated with a protective immune response of the host at the time of Mtb infection [43,44]. Among the cytokines secreted from functional T cells, the simultaneous secretion of IFN-γ, TNF-α, and IL-2 from Th1 CD4^+^ T cells is a crucial component of the protective response against Mtb infection [45]. Therefore, we analyzed a combination of CD4^+^ and CD8^+^ multifunctional T cells in the lungs and spleens that secrete IFN-γ, TNF-α, and IL-2. Live individual T cells were gated on CD4^+^ or CD8^+^, and triple-positive (IFN-γ^+^TNF-α^+^IL-2^+^), double-positive (IFN-γ^+^TNF-α^+^, IFN-γ^+^IL-2^+^, TNF-α^+^IL-2^+^), and single-positive (IFN-γ^+^, TNF-α^+^, IL-2^+^) T cells were analyzed among the CD62L^lo^CD44^hi^ T cells (Figure S3). Four weeks after the final immunizations, the splenocytes and lung cells of the BCG-, CAF01/NG-AG85A-, and CAF01/G-85A-immunized groups and the CAF01 adjuvant control group were restimulated with NG- or G-Ag85A. The CAF01/G-Ag85A-immunized group tended to show a similar but higher frequency of triple- and double-positive cytokine-secreting CD4^+^ T cells in the lung than the CAF01/NG-Ag85A-immunized group (Figure 4a,c; left panel). Triple- and double-positive cytokine-secreting CD8^+^ T cells were barely detected in the lung (Figure 4b,c; right panel). Unlike the lung results, the CAF01/NG-Ag85A- and CAF01/G-Ag85A-immunized groups tended to show a similar frequency of triple- and double-positive cytokine-secreting spleen CD4^+^ T cells (Figure S4). These results indicated that NG- and G-Ag85A have distinctive immunogenic abilities.

### 3.5. G-Ag85A Immunization Provides Superior Protection Compared With NG-Ag85A in Terms of Bacterial Burden and Pathological Lung Lesions 

**Figure 5:**
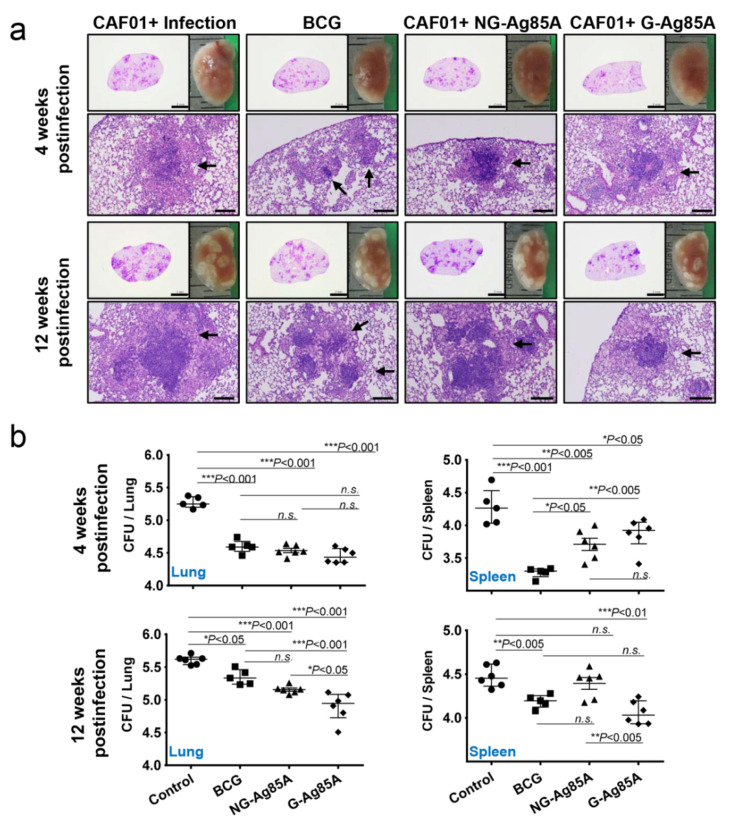
Protective efficacy of plant-produced G-Ag85A and bacteria-produced NG-Ag85A immunization against Mtb HN878 infection. Four weeks after the final immunization, the mice were challenged with 70 CFUs of the Mtb HN878 strain via aerosol. (**a**) The superior lobes of the right lung of each immunized mouse were analyzed using H&E staining, and representative lung lobes were depicted as gross images at 4 and 12 weeks after Mtb HN878 infection (10X: Scale bar  =  2.0 mm, 100X: Scale bar  =  0.2 mm). (**b**) The CFUs in the lungs and spleens of each group were analyzed by culturing lung and spleen homogenates and enumerating the bacteria. The data are presented as the medians ± IQR log_10_CFU/organ (5–6 mice per group at each designated time point), and the levels of the significance of the differences obtained in the comparisons among the samples were determined by one-way ANOVA followed by Dunnett’s test. A value of *p <* 0.05 was considered statistically significant. * *p <* 0.05, ** *p <* 0.01, and *** *p <* 0.001; *n.s.*, not significant.

After immunization, we challenged the mice with the hypervirulent Mtb strain HN878 to assess the protective effect of NG- and G-Ag85A. To compare the protective efficacy of vaccinations, the gross pathology and hematoxylin and eosin (H&E) staining of lung sections from all groups were analyzed (Figure 5a). Four weeks after infection, every Mtb HN878-infected group displayed mild peribronchiolar inflammatory lesions, but there was no significant difference in the extent of granulomatous inflammation in any group of lungs. Twelve weeks after infection, every immunized group showed controlled pathological lesions compared to the infection control group. Excessive, irregular, and advanced granulomatous inflammation of lungs from the infection control and CAF01/NG-Ag85A-immunized groups were shown at 12 weeks postinfection, whereas small, rounded and contained lesions were found in the lungs of mice vaccinated with BCG and CAF01/G-Ag85A (Figure 5a, arrows). Similar to the results based on lesion appearance, controlled bacterial growth was observed in the lungs of all the immunized groups (BCG, CAF01/NG-Ag85A, and CAF01/G-Ag85A) (Figure 5b, upper left). Although every immunized group showed limited bacterial growth in the spleen, only the BCG-immunized group exhibited a significant decrease in the bacterial burden at 4 weeks after infection (Figure 5b, upper right). At 12 weeks postinfection, the G-Ag85A group exhibited better protective efficacy in the lungs and spleens compared with all the other groups (Figure 5b, lower). These results indicated that glycosylation endowed Ag85A with better and prolonged protection against infection with the Mtb strain HN878 in a mouse model.

### 3.6. Immunization with G-Ag85A Induced a More Protective T cell Response against Infection with a Virulent Mtb HN878 Strain than Did Immunization with NG-Ag85A

**Figure 6:**
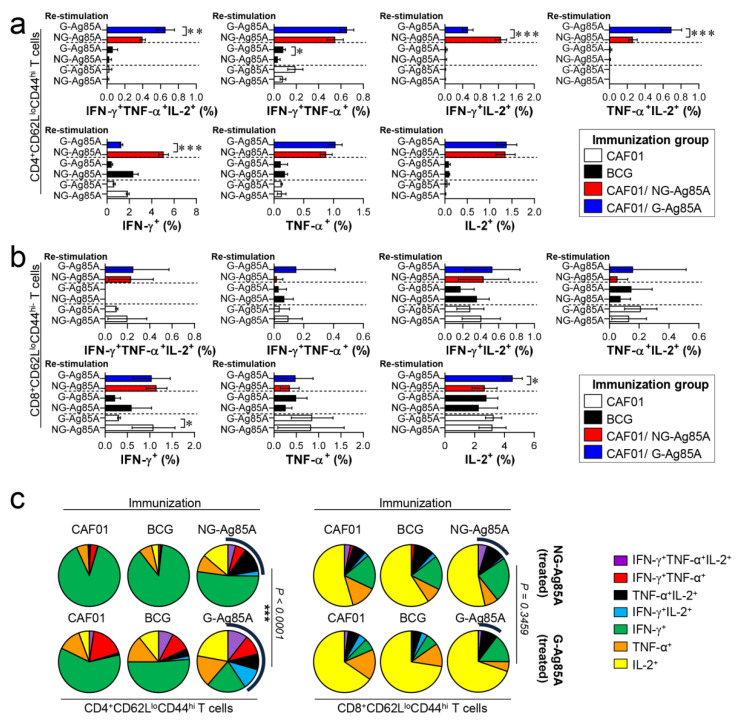
Ag-specific multifunctional T cell responses in plant-produced G-Ag85A- and bacteria-produced NG-Ag85A-immunized mice in the early phase of Mtb HN878 infection. Four weeks postinfection, the mice from each group were euthanized, and their lung cells (1 × 10^6^ cells) were restimulated ex vivo with NG-Ag85A or G-Ag85A (2.5 μg/mL). The percentages of Ag-specific CD4^+^CD62L^lo^CD44^hi^ and CD8^+^CD62L^lo^CD44^hi^ T cells producing IFN-γ, TNF-α, and/or IL-2 among the cells isolated from the lungs of each group of mice were analyzed via flow cytometry by gating the cells into CD4^+^ (**a**) and CD8^+^ (**b**) T cells. The data are presented as the means ± SDs from 5–6 mice in each group. One-way ANOVA was used to determine the significance of the differences. * *p* < 0.05, ** *p <* 0.01, and *** *p <* 0.001. (**c**) The mean frequencies of cells coexpressing IFN-γ, TNF-α, and/or IL-2 are shown in the pie charts. The arcs around the pie charts indicate the percentage of T cells that produced multiple cytokines. Unpaired *t*-tests were used to determine the significance of the differences in the percentage of polyfunctional T cells between the G-Ag85A- and NG-Ag85A-immunized groups.

Based on the immunization results, we subsequently evaluated whether the Th1 immune response generated by immunization with G- or NG-Ag85A continued to secrete IFN-γ to produce effective multifunctional T cells after challenge with HN878 in the spleen and lungs over time. For this analysis, the adjuvant control group and every immunized group were challenged with Mtb and sacrificed at 4 weeks and 12 weeks postinfection, and the lung cells and splenocytes were stimulated with the immunized proteins G- or NG-Ag85A. Compared with the NG-Ag85A-immunized group, the G-Ag85A-immunized group showed only approximately 0.85-fold secretion of IFN-γ in the lung at 4 weeks postinfection but displayed 1.4-fold secretion at 12 weeks postinfection (Supplemental Figure S5). Interestingly, IFN-γ secretion in the spleen was significantly lower in the G-Ag85A-treated group than in the NG-Ag85A-treated group at both 4 and 12 weeks postinfection (Supplemental Figure S5). To analyze the T cells that secrete cytokines, we stained spleen and lung cells in the same manner as in Figure 4. Compared with the CAF01/NG-Ag85A-immunized group, the CAF01/G-Ag85A-immunized group showed an increased percentage of triple-positive multifunctional CD4^+^ T cells and TNF-α^+^IL-2^+^ double-positive cytokine-secreting CD4^+^ T cells in the lung (Figure 6a), and no differences in the percentage of triple-positive or double-positive CD8^+^ T cells were found between the G-Ag85A- and NG-Ag85A-immunized groups (Figure 6b). Compared to the CAF01/G-Ag85A-immunized group, the CAF01/NG-Ag85A-immunized group had twice as many IFN-γ^+^IL2^+^ CD4^+^ T cells (NG-Ag85A: 1.26%, G-Ag85A: 0.52%) and 4 times as many IFN-γ^+^ CD4^+^ T cells (NG-Ag85A: 5.07%, G-Ag85A: 1.28%) as the CAF01/G-Ag85A-immunized group. However, the percentages of every combination of cytokine-secreting CD4^+^ T cells were balanced in the CAF01/G-Ag85A-immunized group but not in the CAF01/NG-Ag85A-immunized group, and the overall frequency of triple-positive and double-positive CD4^+^ T cells was also higher in the CAF01/G-Ag85A-immunized group than in the CAF01/NG-Ag85A-immunized group (Figure 6c). 

**Figure 7:**
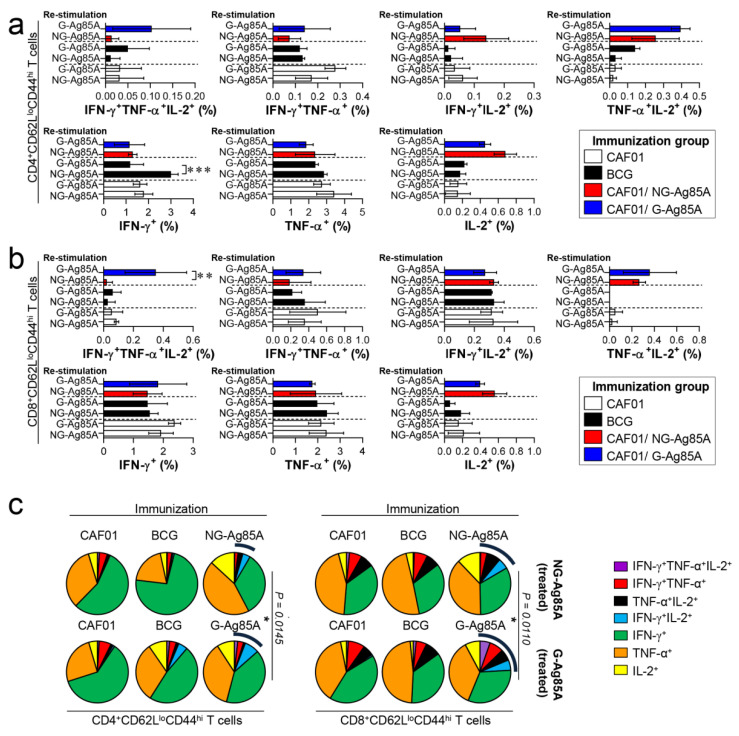
Ag-specific multifunctional T cell responses in plant-produced G-Ag85A- and bacteria-produced NG-Ag85A-immunized mice in the late phase of Mtb HN878 infection. Twelve weeks postinfection, the mice from each group were euthanized, and their lung cells (1 × 10^6^ cells) were restimulated ex vivo with NG-Ag85A or G-Ag85A (2.5 μg/mL). The percentages of Ag-specific CD4^+^CD62L^lo^CD44^hi^ and CD8^+^CD62L^lo^CD44^hi^ T cells producing IFN-γ, TNF-α, and/or IL-2 among the cells isolated from the lungs of each group of mice were analyzed via flow cytometry by gating the cells into CD4^+^ (**a**) and CD8^+^ (**b**) T cells. The data are presented as the means ± SDs from 5–6 mice in each group. One-way ANOVA was used to determine the significance of the differences. * *p <* 0.05, ** *p <* 0.01, and *** *p <* 0.001. (**c**) The mean frequencies of cells coexpressing IFN-γ, TNF-α, and/or IL-2 are shown in the pie charts. The arcs around the pie charts indicate the percentage of T cells that produced multiple cytokines. Unpaired *t*-tests were used to determine the significance of the differences in the percentage of polyfunctional T cells between the G-Ag85A- and NG-Ag85A-immunized groups.

These Ag-specific CD4^+^ T cell responses in the CAF01/G-Ag85A-immunized group waned at 12 weeks postinfection (Figure 7a). In contrast to the Ag-specific CD4^+^ T cell response, the CD8^+^ T cell responses of the G- and NG-Ag85A-immunized groups in the lung were similar at 4 weeks postinfection, but the G-Ag85A-immunized group exhibited more triple-positive multifunctional T cells (NG-Ag85A: 0.02%, G-Ag85A: 0.35%) and a higher overall portion of polyfunctional T cells than did the NG-Ag85A-immunized group (Figure 7b,c). The T cell responses of the spleen were similar to those of the lungs, and the G-Ag85A-immunized group showed a more potent and balanced combination of CD4^+^ T cell responses than did the NG-Ag85A immunized group at 4 but not 12 weeks postinfection (Supplemental Figures S6 and S7). These results indicated that immunization with G-Ag85A could induce a more protective CD4^+^ T cell response against infection with a virulent Mtb strain than could immunization with NG-Ag85A.

## 4. Discussion

Although many TB vaccine Ags, such as Ag85B and ESAT-6, have been produced in different transgenic plant systems [19,20,46], substantial protective vaccine efficacy has not been fully achieved after virulent Mtb challenge in preclinical animal models. To the best of our knowledge, the current study provides the first clear demonstration that the well-known TB vaccine Ag candidate Ag85A, after its glycosylation and production in a plant system, exhibits better potential for long-term protection against Mtb infection than does NG-Ag85A purified from *E. coli*.

During the production of Mtb Ags in plants, one of the most crucial steps is an easy and efficient purification, unless the aim is the oral administration of plants expressing Ags. In fact, in most cases of recombinant protein production, the purification process is the rate-limiting step. Thus, the development of a cost-effective purification system is important. For easy purification, two important components are an affinity tag and a low-cost matrix for the binding of the tag. CBM3 is a cellulose-binding domain of *Clostridium thermocellum* that shows high affinity for MCC [35,47]. In the current study, we used CBM3 as an affinity tag, and the CBM3-Ag85A fusion protein was specifically and strongly bound to MCC beads (Figure 1b). After binding to MCC beads, Ag85A was released from the recombinant Ag85A fusion protein by proteolytic cleavage using enterokinase. Enterokinase specifically recognizes the specific sequence Asp-Asp-Asp-Asp-Lys and cleaves at a site in the C terminus, thereby leaving no additional residues on the C-terminally fused target protein [40]. The 35-kDa protein species was larger than the calculated molecular mass, which indicated that Ag85A was posttranslationally modified by the plant system. Indeed, treatment with Endo-H or PNGase F decreased the size, which indicated that the protein was N-glycosylated (Figure 1d). In fact, Mtb is considered to undergo only O-glycosylation [48]; however, very recently, Birhanu et al. investigated 2944 glycosylation events discovered in 1325 proteins and reported that approximately 17% of the glycosylation sites were N-glycosylated [49]. These data provide the first report of N-linked protein glycosylation in Mtb, including Ag85B and Ag85C, which have similar structures and functions to those of Ag85A. In addition, Spencer et al. demonstrated that the Ag85A produced in chick embryo fibroblasts using a poxvirus shuttle vector had an additional molecular weight of approximately 45 kDa due to N-linked glycosylation [41]. Although the glycosylation of Ag85A has not been experimentally verified, several (at least three) N-linked glycosylation sites have been predicted in Ag85A (http://www.cbs.dtu.dk/services/NetNGlyc/). Because *E. coli-*produced Ag85A had no modifications, such as glycosylation or lipidation, plant-produced G-Ag85A might induce immune responses that are different from those induced by *E. coli*-produced Ag85A [50].

In this study, G-Ag85A expressed in *N. benthamiana* induced a greater Ag-specific IFN-γ response in the lung cells of infected mice than that induced by NG-Ag85A expressed in *E. coli* (Figure 2). These results suggested that G-Ag85A might be better recognized by the host immune system than NG-Ag85A and might exhibit more effective vaccine Ag potential against Mtb. The position of glycans on proteins [51] or the length and size of carbohydrate chains [52,53] could affect the processing of Ags because glycosylation could affect the protein cleavage pattern by proteases, and glycosylation could change immunodominant T cell determinants or epitope recognition [54]. Therefore, these alterations induced by glycosylation could be the reason for the increased recognition of G-Ag85A by Ag-specific immunity compared to NG-Ag85A. In addition, the role of Ag-presenting cells (APCs) is important for the development of a cellular immune response through vaccines. Glycosylation has been shown to be effective as a vaccine or adjuvant because of its ability to alter the response to Ags by signaling through the C-type lectin receptors (CLRs) of APCs, such as dendritic cells (DCs) and macrophages [55,56,57]. These reports have suggested that the different immune responses observed in our study could have resulted from the presence or absence of glycosylation in two identical proteins. In addition, compared with NG-Ag85A-treated BMDCs, G-Ag85A-stimulated BMDCs exhibited increased levels of T cell proliferation and IFN-γ secretion (Figure 2). Thus, glycosylation might give Ag85A the capability to induce a different T cell response through APCs, such as DCs, and this ability could explain the discrepancy in the protective efficacy between G-Ag85A and NG-Ag85A immunization.

We evaluated the protective efficacy of G-Ag85A- and NG-Ag85A immunization against Mtb HN878 in a mouse model. At 4 weeks postinfection, the lung-inflamed lesions or bacterial burdens of lung tissues from both the G-Ag85A- and NG-Ag85A-immunized groups were similar to those of the BCG-immunized group (Figure 5). However, after 12 weeks postinfection, compared with NG-Ag85A-immunized mice, G-Ag85A immunized mice showed mild lung inflammation and limited bacterial burden in lung tissues. Although a clear protective correlation has not been fully proven, the multifunctional Th1 T cell response has been shown to be associated with protection against Mtb infection in mouse models [58,59,60]. In addition, a continuous decrease in the multifunctionality of T cells has been correlated with a decrease in the protection against Mtb infection in a mouse model [45]. Thus, we compared CD4^+^ Th1 T cells that simultaneously secreted two or three cytokines (IFN-γ, TNF-α, and IL-2) following immunization with G-Ag85A or NG-Ag85A. Consequently, the percentage of polyfunctional CD4^+^ T cells that secreted more than one cytokine was higher and more balanced in the G-Ag85A-immunized group than in the NG-Ag85A-immunized group at 4 weeks postinfection (Figure 6). Long-lasting IFN-γ expression (Supplemental Figure S5) was detected in the G-Ag85A-immunized group, and these results support the protective efficacy of G-Ag85A in lung pathology and the reduction in bacterial load.

Although CD4^+^ T cells that produce IFN-γ are considered indispensable for TB protection, the relative importance of CD8^+^ T cells has been underestimated. Recent studies, however, have shown that CD8^+^ T cells contribute to TB [61]. Mtb induces an immune evasion mechanism that inhibits the recognition of Mtb Ag TB10.4 by CD8^+^ T cells during macrophage infection [62]. The depletion of CD8^+^ T cells impaired BCG vaccination-induced immunity and protection against Mtb in a nonhuman primate model [63]. These results suggest that lung polyfunctional CD8^+^ T cells induced by G-Ag85A immunization might play an important role in the protection against Mtb infection, at least until 12 weeks postinfection in our study (Figure 7b,c). In addition, a greater difference between G-Ag85A- and NG-Ag85A-stimulated BMDCs in terms of their ability to promote T cell proliferation was observed in CD8^+^ T cells than in CD4^+^ T cells. These results indicate that the glycosylation of Ag85A resulted in activation of the CD8^+^ T cell response and might help explain the protective effect of G-Ag85A immunization observed at 12 weeks postinfection.

In our study, the effects of G-Ag85A and NG-Ag85A immunization did not exhibit any differences at 4 weeks postinfection, but G-Ag85A immunization, unlike NG-Ag85A immunization, exhibited protective effects in the lung and spleen at 12 weeks postinfection (Figure 5). However, these results did not correlate with the differences in the Ag-specific multifunctional CD4^+^ T cell response at 4 weeks postinfection observed between G-Ag85A and NG-Ag85A immunization. This discrepancy is thought to be the result of the snapshots provided by only studying the time points of 4 and 12 weeks postinfection, and the protective CD4^+^ T cell response that developed up to 4 weeks postinfection appears to affect later time points (12 weeks postinfection) rather than earlier time points. In addition, the CD8^+^ T cell response induced by G-Ag85A immunization at 12 weeks postinfection involved a higher multifunctional CD8^+^ T cell response than that induced by NG-Ag85A immunization, which correlated with reductions in the bacterial load and lung inflammation. Notably, the synergy between CD4^+^ and CD8^+^ T cells suggests that a vaccine that induces both T cell subsets has the best opportunity to prevent TB. Recently, Moguche et al. demonstrated differential responses of CD4^+^ T cells to two leading TB vaccine Ags, Ag85B and ESAT-6, in a mouse model [64]. They reported that the functional exhaustion induced by chronic antigenic stimulation-driven CD4^+^ T cells with a substantial proportion of KLRG1^+^ cells restricts the protective ability of CD4^+^ T cells to recognize ESAT-6, whereas Ag85B-specific T cells exhibit a limited ability to control Mtb infection by reducing Ag expression during persistent Mtb infection. These results suggest that different vaccination strategies will be required to achieve optimal protection mediated by the recognition of Ags expressed at distinct stages of Mtb infection by T cells. Similar to Ag85B, Ag85A is exclusively expressed during the early infection phase, but its expression is reduced during the late phase of Mtb infection. Interestingly, the maintenance of multifunctional Th1 T cell responses and the switching of CD4^+^ T cells to CD8^+^ T cells is associated with the long-term protective efficacy of G-Ag85A immunization in mice. Although we are unable to define how this switching occurred in the current study, our data have important implications for the rational design of TB vaccines tailored to optimize the protection conferred by specific CD4^+^ T cells that recognize Ags expressed at distinct stages of Mtb infection.

## 5. Conclusions

Compared with NG-Ag85A, G-Ag85A induced a strong IFN-γ response in the lung cells of Mtb-challenged mice, and G-Ag85A-matured BMDCs resulted in enhanced T cell proliferation with IFN-γ secretion compared to NG-Ag85A-matured BMDCs. In addition, vaccination with G-Ag85A induced robust, durable, Ag-specific, and balanced Th1 T cell responses, and ultimately conferred enhanced protection against Mtb HN878. However, the association between Ag glycosylation and the immunological responses induced by G-Ag85A and NG-Ag85A has not yet been clearly established. Therefore, further investigations with glycosylation of other Mtb Ags are necessary to confirm this relationship. 

Collectively, our results suggest that post-translationally modified Ag85A, which might mimic the native form of Ag85A produced in a plant system, has high potential as a vaccine Ag by generating balanced Th1 T cell responses with multifunctional capacities. In addition, vaccination through the combination of G-Ag85A with other Ags or its application as a BCG booster could be an effective strategy to increase vaccine efficacy, which might provide new opportunities for the future development of anti-TB vaccines.

## Supplementary Materials

The following are available online at https://www.mdpi.com/2076-393X/8/2/189/s1, Figure S1: Confirmation of glycosylated Ag85A expressed in *N. benthamiana*. Figure S2: Deglycosylation of G-Ag85A and NG-Ag85A. Figure S3: Gating strategy for the analysis of multifunctional T cells in the lungs and spleens. Figure S4: Induction of Ag-specific multifunctional T cells in the spleens of mice immunized with G- and NG-Ag85A. Figure S5: Comparison of the IFN-γ producing capabilities of lung and spleen cells from G-Ag85A- and NG-Ag85A-immunized mice at 4 weeks and 12 weeks postchallenge with Mtb. Figure S6: Ag-specific multifunctional T cell responses in the spleens of G-Ag85A- and NG-Ag85A-immunized mice in the early phase of Mtb HN878 infection. Figure S7: Ag-specific multifunctional T cell responses in the spleens of G-Ag85A- and NG-Ag85A-immunized mice in the late phase of Mtb HN878 infection. Table S1: Nucleotide sequences of the primers used in this study.
