# Supplementary material for: Plant-Produced N-glycosylated Ag85A Exhibits Enhanced Vaccine Efficacy Against Mycobacterium tuberculosis HN878 Through Balanced Multifunctional Th1 T Cell Immunity
Source: Vaccines (Basel). 2020 Apr 18;8(2):189. doi: 10.3390/vaccines8020189 (PMC7349862; doi:10.3390/vaccines8020189)
Supplement: Supplementary file 1 [file vaccines-08-00189-s001.pdf]

Supplemental Material

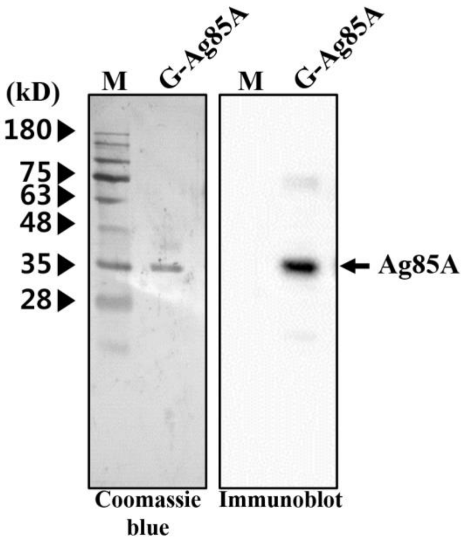

Figure S1. Confirmation of glycosylated Ag85A expressed in *N. benthamiana*. Ag85A was successfully produced in *N. benthamiana*. The purified Ag85A protein was subjected to SDS-PAGE, and the gels were stained with Coomassie brilliant blue. The purified Ag85A was subjected to western blotting analysis with an anti-Ag85A antibody (Lane M, molecular weight markers; Lane G-Ag85A, purified Ag85A).

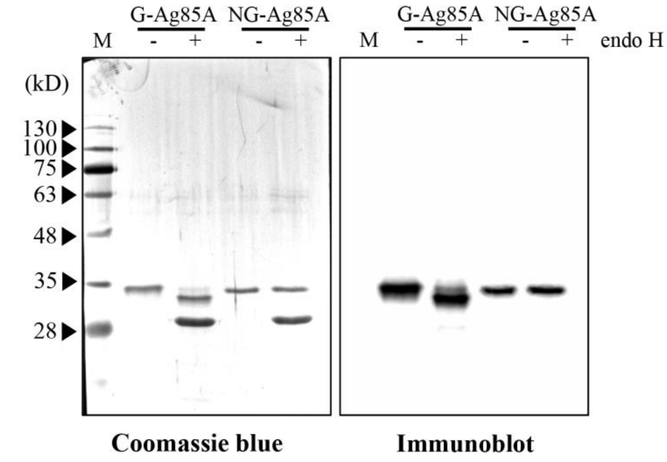

Figure S2. Deglycosylation of G-Ag85A and NG-Ag85A. Purified G-Ag85A from *N. benthamiana* and NG-Ag85A from *E. coli* were treated with Endo-H and subjected to SDS-PAGE together with untreated G-Ag85A and NG-Ag85A, and then to western blot analysis using an anti-Ag85A antibody (Lane M, molecular weight markers).

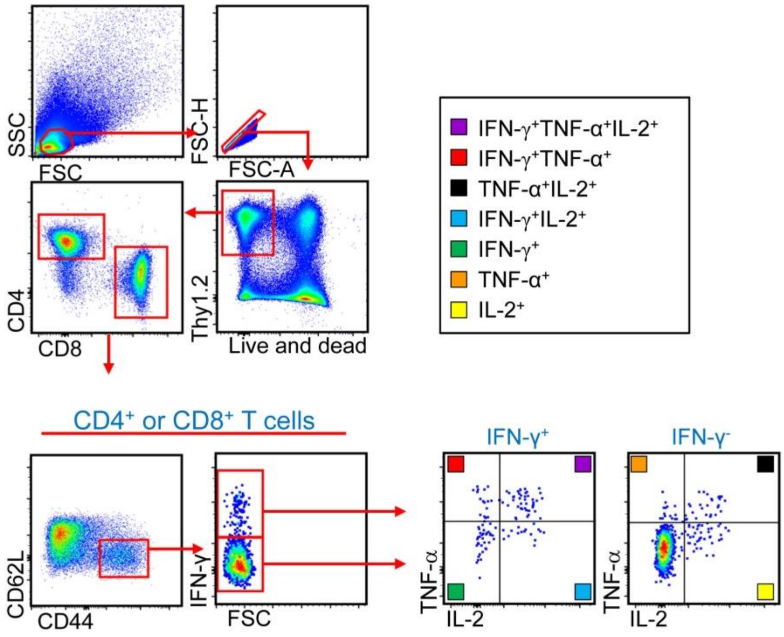

Figure S3. Gating strategy for the analysis of multifunctional T cells in the lungs and spleens. The lymphocyte population was first gated based on their characteristic forward scatter (FSC) and side scatter (SSC) patterns. Single cells were gated based on equivalent FSC height (FSC-H) and FSC area (FSC-A) values to exclude doublets and large cell aggregates. For the analysis of living lymphocytes, the single cell-gated lymphocytes were stained with live and dead stains and Thy1.2 and then gated into $CD4^+$ or $CD8^+$ T cells. $CD62L^{lo}$ and $CD44^{hi}$ cells were gated to profile the T cells producing various cytokines (combinations of IFN-γ, TNF-α, and IL-2).

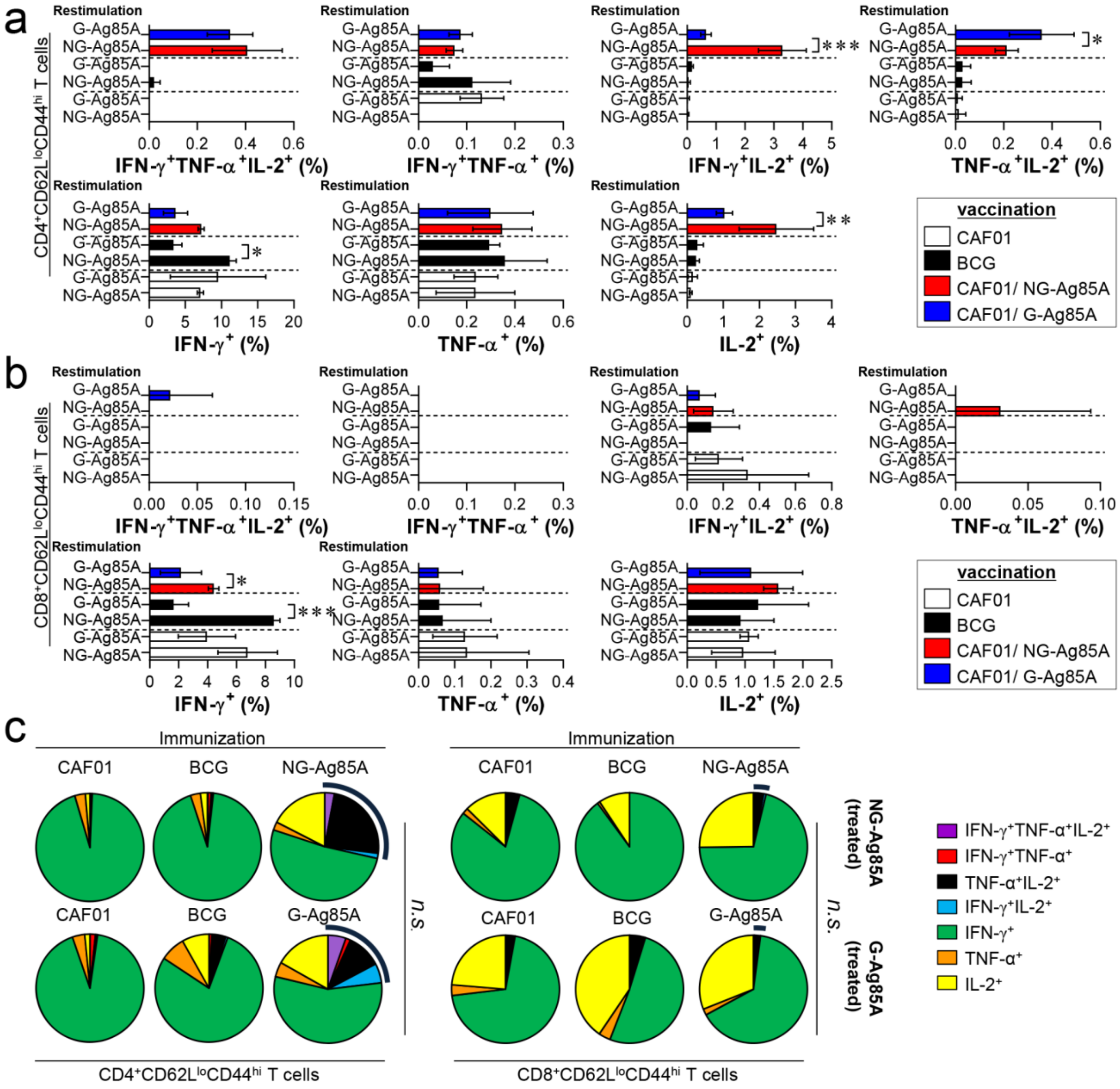

Figure S4. Induction of Ag-specific multifunctional T cells in the spleens of mice immunized with G- and NG-Ag85A. Four weeks after the final immunization, the mice from each group (n = 5–6) were sacrificed, and their spleen cells (2×10^6 cells) were restimulated ex vivo with NG- or G-Ag85A (2.5 μg/mL). The percentages of Ag-specific $CD4^+CD62L^{lo}CD44^{hi}$ and $CD8^+CD62L^{lo}CD44^{hi}$ T cells producing IFN-γ, TNF-α, and/or IL-2 among the cells isolated from the lungs of each group of mice were analyzed via flow cytometry by gating the cells into $CD4^+$ (**a**) and $CD8^+$ (**b**) T cells. The data are presented as the means ± SDs from five to six mice in each group. One-way ANOVA was used to determine the significance of the differences. * $p < 0.05$, ** $p < 0.01$, and *** $p < 0.001$. (**c**) The mean frequencies of cells coexpressing IFN-γ, TNF-α, and/or IL-2 are shown in the pie charts. The arcs around the pie charts indicate the percentage of T cells that produced multiple cytokines. Unpaired t-tests were used to determine the significance of the differences in the percentages of poly-functional T cells between the G-Ag85A- and NG-Ag85A-immunized groups. n.s., not significant.

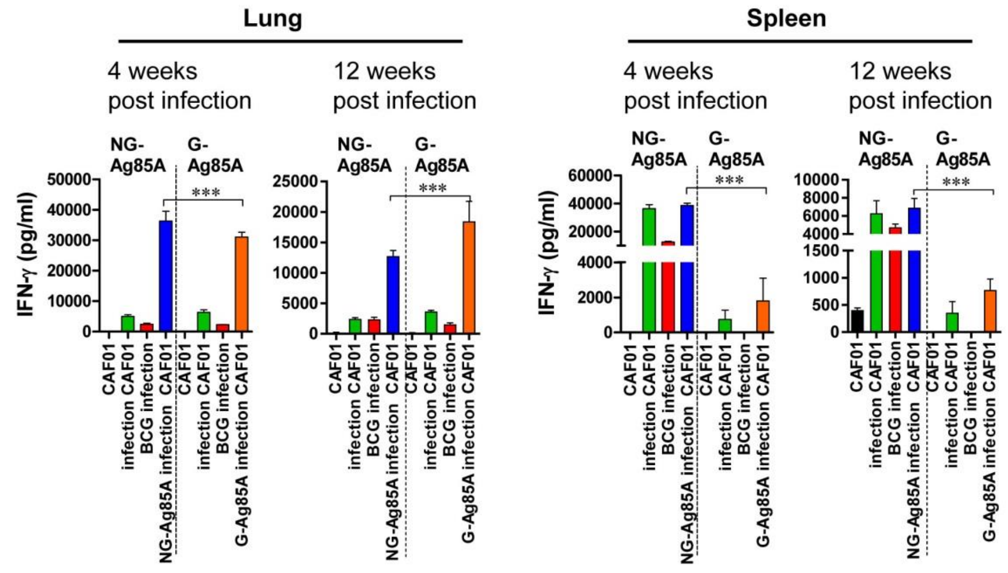

Figure S5. Comparison of the IFN-γ producing capabilities of lung and spleen cells from G-Ag85A- and NG-Ag85A-immunized mice at 4 weeks and 12 weeks post-challenge with Mtb. Four and 12 weeks post-infection, the mice from each group (n = 5–6) were sacrificed, and their lungs (1×10^6 cells) were plated on a microtiter plate and restimulated with NG- or G-Ag85A (2.5 μg/mL) for 12 h at 37 °C. The IFN-γ levels in the supernatant of the incubated lung and spleen cells were detected by ELISA. The data are presented as the means ± SDs from five to six mice in each group. One-way ANOVA was used to determine the significance of the differences. *** $p < 0.001$.

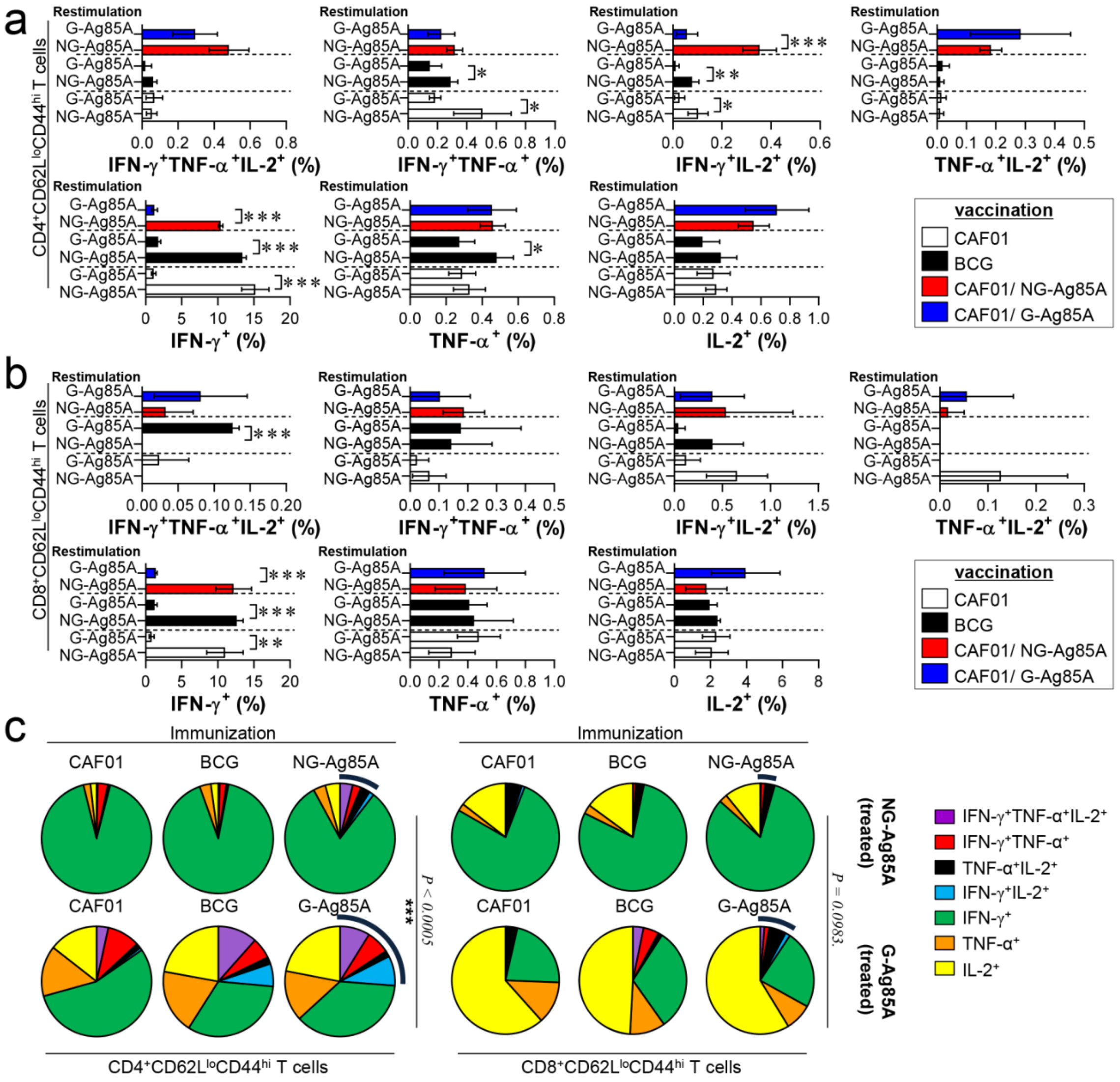

Figure S6. Ag-specific multifunctional T cell responses in the spleens of G-Ag85A- and NG-Ag85A-immunized mice in the early phase of Mtb HN878 infection. Four weeks post-infection, the mice from each group were euthanized, and their spleen cells (2 × 106 cells) were restimulated ex vivo with NG-Ag85A or G-Ag85A (2.5 μg/mL). The percentages of Ag-specific $CD4^{+}CD62L^{lo}CD44^{hi}$ and $CD8^{+}CD62L^{lo}CD44^{hi}$ T cells producing IFN-γ, TNF-α, and/or IL-2 among the cells isolated from the lungs of each group of mice were analyzed via flow cytometry by gating the cells in to $CD4^{+}$ (**a**) and $CD8^{+}$ (**b**) T cells. The data are presented as the means ± SDs from five to six mice in each group. One-way ANOVA was used to determine the significance of the differences. * $p < 0.05$, ** $p < 0.01$, and *** $p < 0.001$. (**c**) The mean frequencies of cells coexpressing IFN-γ, TNF-α, and/or IL-2 are shown in the pie charts. The arcs around the pie charts indicate the percentage of T cells that produced multiple cytokines. Unpaired t-tests were used to determine the significance of the differences in the percentage of poly-functional T cells between the G-Ag85A- and NG-Ag85A-immunized groups.

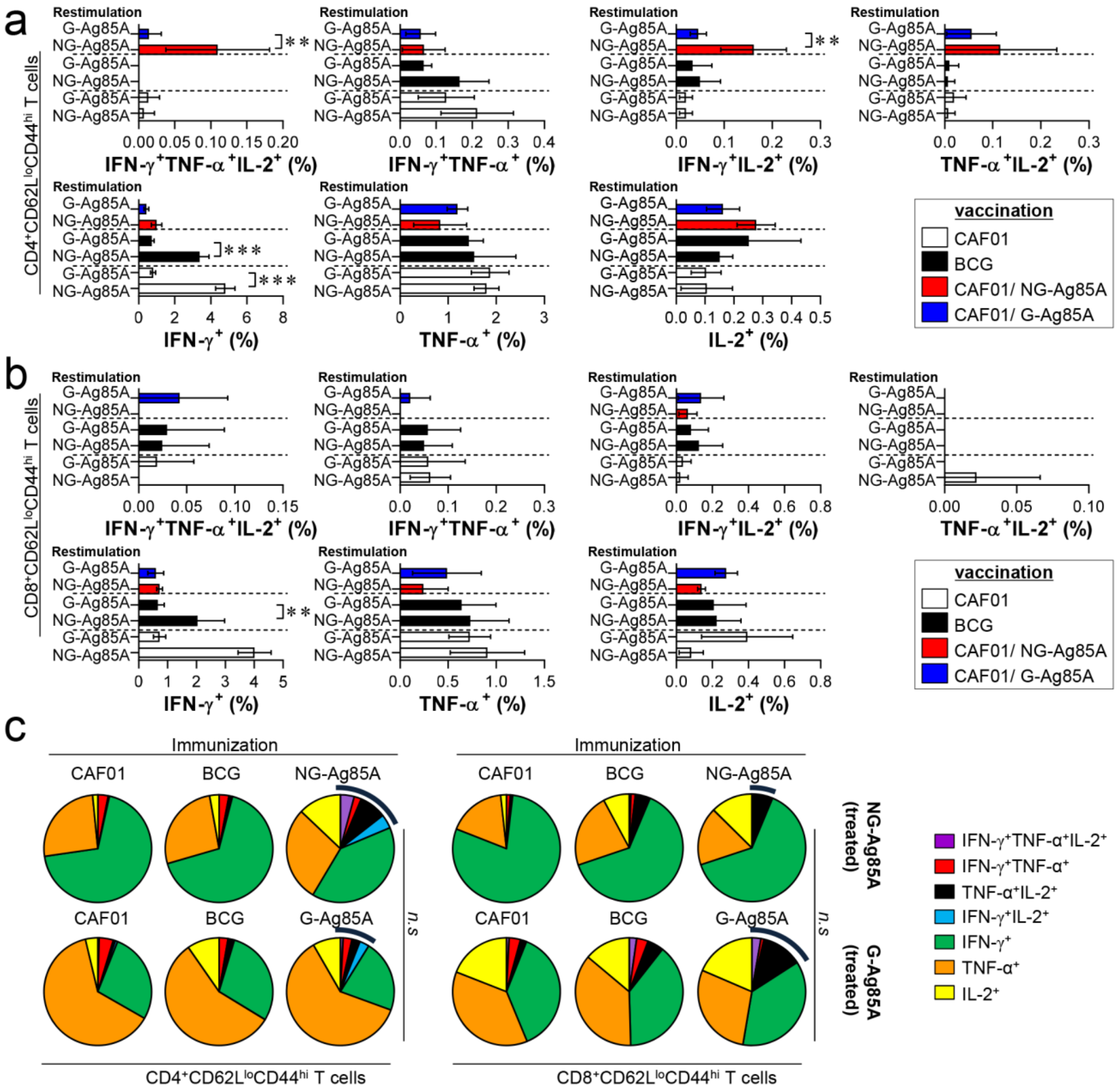

Figure S7. Ag-specific multifunctional T cell responses in the spleens of G-Ag85A- and NG-Ag85A-immunized mice in the late phase of Mtb HN878 infection. Twelve weeks post-infection, the mice from each group were euthanized, and their spleen cells (2 × 10^6 cells) were restimulated ex vivo with NG-Ag85A or G-Ag85A (2.5 μg/mL). The percentages of Ag-specific $CD4^+CD62L^{lo}CD44^{hi}$ and $CD8^+CD62L^{lo}CD44^{hi}$ T cells producing IFN-γ, TNF-α, and/or IL-2 among the cells isolated from the lungs of each group of mice were analyzed via flow cytometry by gating the cells into $CD4^+$ (**a**) and $CD8^+$ (**b**) T cells. The data are presented as the means ± SDs from five to six mice in each group. One-way ANOVA was used to determine the significance of the differences. * $p < 0.05$, ** $p < 0.01$, and *** $p < 0.001$. (**c**) The mean frequencies of cells coexpressing IFN-γ, TNF-α, and/or IL-2 are shown in pie charts. The arcs around the pie charts indicate the percentage of T cells that produced multiple cytokines. Unpaired t-tests were used to determine the significance of the differences in the percentages of poly-functional T cells between the G-Ag85A- and NG-Ag85A-immunized groups. n.s., not significant.

Table S1. Nucleotide sequences of the primers used in this study

Primer Name	Sequence (5′ to 3′)
Ag85A-F	cccgggatgacgacgataagtttagccggcctggcctgcctgtg
Ag85A-R	gagctcatcgtgggcgccctgaggagcagggccggt
CBM3-F	ggatcctctacccatacgatgttccagattacgctccggtatcaggtaaccttaaggtg

CBM3-R	cccgggctttcgctgcagcttccttagcggctgcctcaccaggttcctttccccacactag

F: forward primer. R: reverse primer.
